# The HER3 pathway as a potential target for inhibition in patients with biliary tract cancers

**DOI:** 10.1371/journal.pone.0206007

**Published:** 2018-10-18

**Authors:** Angela Lamarca, Salvatore Galdy, Jorge Barriuso, Sharzad Moghadam, Elizabeth Beckett, Jane Rogan, Alison Backen, Catherine Billington, Mairéad G. McNamara, Richard A. Hubner, Angela Cramer, Juan W. Valle

**Affiliations:** 1 Department of Medical Oncology, The Christie NHS Foundation Trust, Manchester, United Kingdom; 2 Unit of Gastrointestinal Medical Oncology and Neuroendocrine Tumours, European Institute of Oncology, Milan, Italy; 3 Division of Cancer Sciences, School of Medical Sciences, Faculty of Biology, Medicine and Health, University of Manchester, Manchester, United Kingdom; 4 Manchester Cancer Research Centre Biobank, University of Manchester, Manchester, United Kingdom; 5 The Christie Pathology Partnership, The Christie NHS Foundation Trust, Manchester, United Kingdom; University of South Alabama Mitchell Cancer Institute, UNITED STATES

## Abstract

**Introduction:**

Expression of human epidermal growth factor receptor (HER)2 and HER3 have been investigated in small BTC studies using variable scoring systems.

**Methods:**

HER2 and HER3 overexpression/amplification were explored following internationally agreed guidelines using immunohistochemistry (IHC) and fluorescent *in-situ* hybridisation (FISH), respectively. Logistic regression and survival analysis (Kaplan Meier, Log rank test and Cox Regression) were used for statistical analysis.

**Results:**

Sixty-seven eligible patients with Stage I/II (31.3%) or III/IV (68.7%) disease at diagnosis were included. Membrane HER2 overexpression/amplification was identified in 1 patient (1%). HER3 overexpression was predominantly cytoplasmic; the rate of overexpression/amplification of HER3 in membrane and cytoplasm was 16% [ampullary cancer (AMP) (1/13; 8%), gallbladder cancer (GBC) (1/10; 10%), intra-hepatic cholangiocarcinoma (ICC) (6/26; 23%), extra-hepatic cholangiocarcinoma (ECC) (3/18; 17%)] and 24% [AMP (1/13; 8%), GBC (1/10; 10%), ICC (10/26; 38%), ECC (4/18; 22%)], respectively.

**Conclusions:**

A significant subset of patients with BTC expressed HER3. Inhibition of HER3 warrants further investigation. A better understanding of the downstream effects of HER3 in BTC requires further mechanistic investigations to identify new biomarkers and improve patient selection for future clinical trials.

## Introduction

### Biliary tract cancer

Cancers of the biliary tract comprise cholangiocarcinoma (CC), gallbladder cancer (GBC) and ampullary cancer (AMP); all have a poor-prognosis with a five year overall survival (OS) of 18% when all stages are analysed together [1]. Current standard-of-care first-line treatment for patients with advanced biliary tract cancer (BTC) is cisplatin-gemcitabine chemotherapy, based on the UK ABC-02 clinical trial [2]. This study reported a median OS of 11.7 months for patients treated with cisplatin and gemcitabine, compared to 8.1 months for patients treated with gemcitabine alone (P<0.001). Benefit from second-line chemotherapy remains unclear [3], however ongoing studies are currently exploring the role of chemotherapy in this setting [4].

There has been an interest in identification of potential targets in combination with a precision medicine approach for patients with BTC [5]. Unfortunately, none of the studies completed to date have improved the current standard of care [6,7]. Results from studies targeting Fibroblast Growth Factor Receptor (FGFR) [8,9,10] and isocitrate dehydrogenase (IDH) [11] (predominantly in intra-hepatic cholangiocarcinoma (ICC)) show promise and are expected to change the treatment paradigm for patients with ICC, although final results of ongoing studies are awaited for confirmation of real benefit (e.g. NCT02428855, NCT02989857, NCT02150967). Adequately-powered randomised controlled trials of novel agents (or combinations of agents) are needed, along with effective biomarkers to allow suitable patient selection or prediction of treatment response.

### HER receptor family

The human epidermal growth factor receptor (HER, also known as ERBB) family consists of four members: epidermal growth factor receptor (EGFR, also termed HER1), HER2, HER3, and HER4 [12]. Binding of ligands leads to the homo-dimer and hetero-dimer formation of these receptor tyrosine kinases [13]. HER2 is a ligand-less receptor that functions as a co-receptor with other HER members; this means HER2 is recruited into HER ligand complexes. HER3 contains an inactive tyrosine kinase domain and forms a heterodimer with HER2 in a ligand-mediated manner [14].

The HER family play a key role in carcinogenesis and disease progression in several human cancers. For example, HER2 is overexpressed in around 20–30% of breast cancer tumours. It is associated with more aggressive disease, higher recurrence rate, and increased mortality [15]. Ligand binding to the extracellular domain results in receptor homo- or hetero-dimerization, a critical step in HER family-mediated signaling. Dimerization leads to the activation of different downstream signaling cascades, including the mitogen-activated protein kinase (MAPK) proliferation pathway and phosphatidylinositol 3-kinase (PI3K)/Protein kinase B (PKB or Akt) pro-survival pathway[16,17,18,19].

HER2 and HER3 pathways may be potential candidates for disruption with treatment for patients with BTC. Studies in BTC cell lines have confirmed this hypothesis [20]. Several rodent ICC studies have developed transgenic animals which constitutively overexpressed HER2 in the epithelium, which triggered and increased risk of development of BTC [21].

### Challenges of HER2 and HER3 staining in biliary tract cancer

Several trials treating patients with BTC with inhibitors of the HER pathway (when HER2 overexpression was identified) have yielded disappointing results [for example lapatinib [22,23], and erlotinib [24]]. Results were more encouraging when targeting mutations, rather than overexpression [neratinib [25]], but this has yet to be repeated in a larger patient cohort. Unfortunately, high-quality data regarding expression and/or amplification rates in BTC is lacking [21]. A systematic review and meta-analysis has investigated the use of HER2 and HER3 as biomarkers in patients with BTC [26]. Forty studies were identified which reported HER2 and/or HER3 membrane protein expression using IHC and/or gene amplification using ISH in tumours of patients with BTC. Studies were classified as ‘high quality’ if IHC overexpression was defined as presence of moderate or strong staining, or ‘low quality’ where ‘any’ expression was considered positive. This highlighted the fact that non-standardised approaches have been adopted in BTC, which made comparison of results challenging. It also became apparent that conclusions were often limited by small study sizes (with combinations of different BTC sub-types such as ICC, extrahepatic cholangiocarcinoma (ECC), gallbladder cancer and ampullary cancer).

This study investigated the prevalence of overexpression and amplification of HER2 and HER3 in patients with BTC, using a standardised approach for IHC and FISH methodology and interpretation. This allows assessment of these potential drug targets in this patient population, which could ultimately be informative for future drug development.

## Material and methods

Patients diagnosed with BTC (including CC, GBC and AMP) with available formalin-fixed paraffin-embedded (FFPE) archival tumour tissue, were eligible. All patients had provided informed consent for tissue storage and subsequent use of their tumour tissue(s) for research purposes. This study was approved by the Manchester Cancer Research Centre BioBank Ethics Committee. Clinical and outcome data were collected from hospital records.

### Analysis of samples

Overexpression of HER2 and HER3 protein was determined by IHC and HER2 and HER3 gene amplification by FISH.

Using the Benchmark Ultra automated staining platform HER2, immunostaining was carried out using the standardised, validated Pathway anti-HER2 (clone 4B5) rabbit monoclonal (Roche, Ventana, Cat No 790–2991) in combination with Ventana ultraView DAB detection kit (Cat No 760–500). HER3 immunostaining was carried out using anti-erbB-3/HER3 (clone 2F12) mouse monoclonal (Millipore, Cat No 05–390), 0.2mg/mL at a dilution 1:150, also coupled with the Ventana ultraView DAB detection kit.

HER2 FISH was carried out using the Leica HER2 FISH system dual colour probe for HER2 (Leica Biosystems, cat No TA9217) and HER3, FISH using the ZytoVision HER3/CEN12 dual colour probe.

### Scoring of IHC

Since no pre-defined guidelines for IHC scoring in BTC were available, HER2 scoring was interpreted following guidelines for both gastric cancers [27,28] and breast cancer [29] (S1 Table). In order to define whether the gastric or the breast criteria were most appropriate for BTC assessment, both scoring systems were utilised for HER2 and compared in a subset of samples. The selected scoring system was used for definition of overexpression of HER2 in the whole series and the same criteria were then used for HER3 scoring.

Only membrane staining was considered when scoring HER2 (as suggested by international guidelines [27,28];[29]), whereas both cytoplasmic and membrane staining were taken into account for the scoring of HER3. Positive and negative controls were used to validate antibody batches used in the analysis. Samples scoring HER2 3+ were regarded as positive, while those scoring IHC0 or IHC1+ were recorded as negative. The same criteria used for HER2 membrane scoring was employed for HER3 membrane scoring. Cases scoring IHC2+ were considered borderline, equivocal and referred for confirmation of both HER2 and HER3 status by FISH, as per previous groups [30]. A further score of IHC0, IHC1+, IHC2+ and IHC3+ was determined for intensity and coverage of cytoplasmic staining.

### Scoring of FISH

Using the dual probe method, ratio of HER2 signal to chromosome 17 centromeric enumeration probe (CEP17) signal, HER2/CEP17, as well as average HER2 copy number were calculated. Similarly, ratio of HER3 signal to chromosome 12 centromeric enumeration probe (CEN12) signal, HER3/CEN12, as well as average HER3 copy number, were calculated.

Tumours with a ratio > 2.0 and/or average HER2 or HER3 copy number > 6.0 were considered positive. Tumours with a ratio <2.0 and average HER2 or HER3 copy number <4.0 were considered negative. Where the ratio was <2.0, but the average HER2 or HER3 copy number was >4.0 and <6.0, the tumour was considered borderline but not amplified/negative.

### Statistical analyses

Sample-size calculation showed that 70 patients were required for this analysis, with a power of 0.91 and an alpha-error of 0.1, in order to confirm a minimal difference of 10% expression (null hypothesis [no clinically relevant expression (predefined as 5% of samples showing positive findings)]; alternative hypothesis [clinically relevant expression (predefined as 15% of samples showing positive findings)].

The student’s t-test and chi-square test were used for analysis, as appropriate. Agreement between scoring systems was calculated by Kappa analysis. Logistic regression was used to identify factors associated with HER2 or HER3 overexpression. Variables with a p-value <0.05 in the univariate analysis were included in the multivariable analysis.

For correlation with clinical outcomes, overall survival (OS) was defined as the time from diagnosis to death (patients alive at the end of follow-up were censored at the date of last follow-up). Progression-free survival (PFS) was calculated for patients treated with first-line palliative chemotherapy as the time between starting palliative chemotherapy and time of progression or death (patients alive and without progression at the end of follow-up were censored at the date of the last follow-up). The Kaplan-Meier method, Log-rank test and Cox Regression (also known as proportional hazards regression) were used to evaluate survival analysis.

Statistical analyses were carried out using Stata version 12.0 (StataCorp, Texas, United States).

### “In silico” analysis.

In order to show data from international consortia in BTC, CbioPortal[31] “in silico” analysis of data available from the genomic consortia including 131 patients was performed (last accessed 20th July 2018).

## Results

Of 167 screened patients between January 2013 and July 2015, 76 FFPE tumour samples were retrieved for quality assessment, and a total of 67 samples were considered eligible (S1 Fig).

### Patient characteristics

Summary of patient characteristics is illustrated in Table 1. Of the sixty-seven patients included, the majority had a diagnosis of CC (65.67%). Twenty-one patients (68.66%) were diagnosed with local stage disease (stage I (7.46%), stage II (23.88%)) and 46 (68.66%) with advanced stage disease (stage III (16.42%), stage IV (51.24%)). All tumours were classified as adenocarcinomas, predominantly moderately-differentiated (55.22%). Median and 95% confidence interval (CI) of baseline CA19.9 (IU/ml), albumin and neutrophil/lymphocyte ratio (NLR) was 43.00 (95% CI 24.42–125.84), 42.00 (41.00–43.00) and 3.41 (2.74–4.05), respectively.

**Table 1:** Baseline characteristics of the 67 eligible patients included in this study.

		N	%
**Age** (years)	Median (95% CI)	65.6 (62.1–69.0)	
**Gender**	Female	35	51.24
	Male	32	47.78
**ECOG performance status**	0	19	28.36
	1	38	56.72
	2	9	13.43
	3	1	1.49
**Primary tumour site**	Cholangiocarcinoma (all)	44	65.67
	*Intra-hepatic CC*	*26*	*38*.*81*
	*Extra-hepatic CC*	*18*	*26*.*87*
	Ampullary cancer	13	19.40
	Gallbladder	10	14.93
**Tumour differentiation**	Well-differentiated	3	4.48
	Moderately-differentiated	37	55.22
	Poorly-differentiated	18	26.87
	Not specified	9	13.43
**Disease stage**	I	5	8
	II	16	24
	III	11	17
	IV	35	51
**Aim of treatment at first diagnosis**	Curative	26	38.81
	Palliative	41	61.19
**Received adjuvant chemotherapy**	Yes*	14	20.90
**Received palliative chemotherapy**	Yes^#^	42	62.69

95%CI: 95% confidence interval; CC: cholangiocarcinoma; ECOG: Eastern Cooperative Oncology Group.

* adjuvant chemotherapy included fluoropyrimidine (8 patients), gemcitabine (3 patients) combination of gemcitabine and capecitabine (3 patients).

^#^ Palliative chemotherapy included: gemcitabine plus platinum (32 patients), gemcitabine single agent (1 patient), Fluoropyrimidine plus platinum (1 patient).

### Recommended IHC criteria

A randomly selected group of twenty-seven samples were analysed for HER2 IHC using both gastric [27,28] and breast [29] criteria. Although agreement between both criteria was high (77.78%, S2 Table), the Kappa index was low (0.2703). In addition, breast criteria tended to underestimate the scoring of tumours, with six patients classified as IHC0 by breast criteria, being upgraded to IHC1+ (five patients) and IHC2+ (one patient) based on gastric assessment. In addition, morphologic similarity between BTC and gastric cancers was also in favour of recommending the gastric criteria for the whole series and for HER3 analysis. Based on these grounds, gastric criteria for IHC assessment was used in the whole population, and for reporting of overexpression of HER2 and HER3 in this study.

### Evaluation of HER2 and HER3

**Fig 1:**
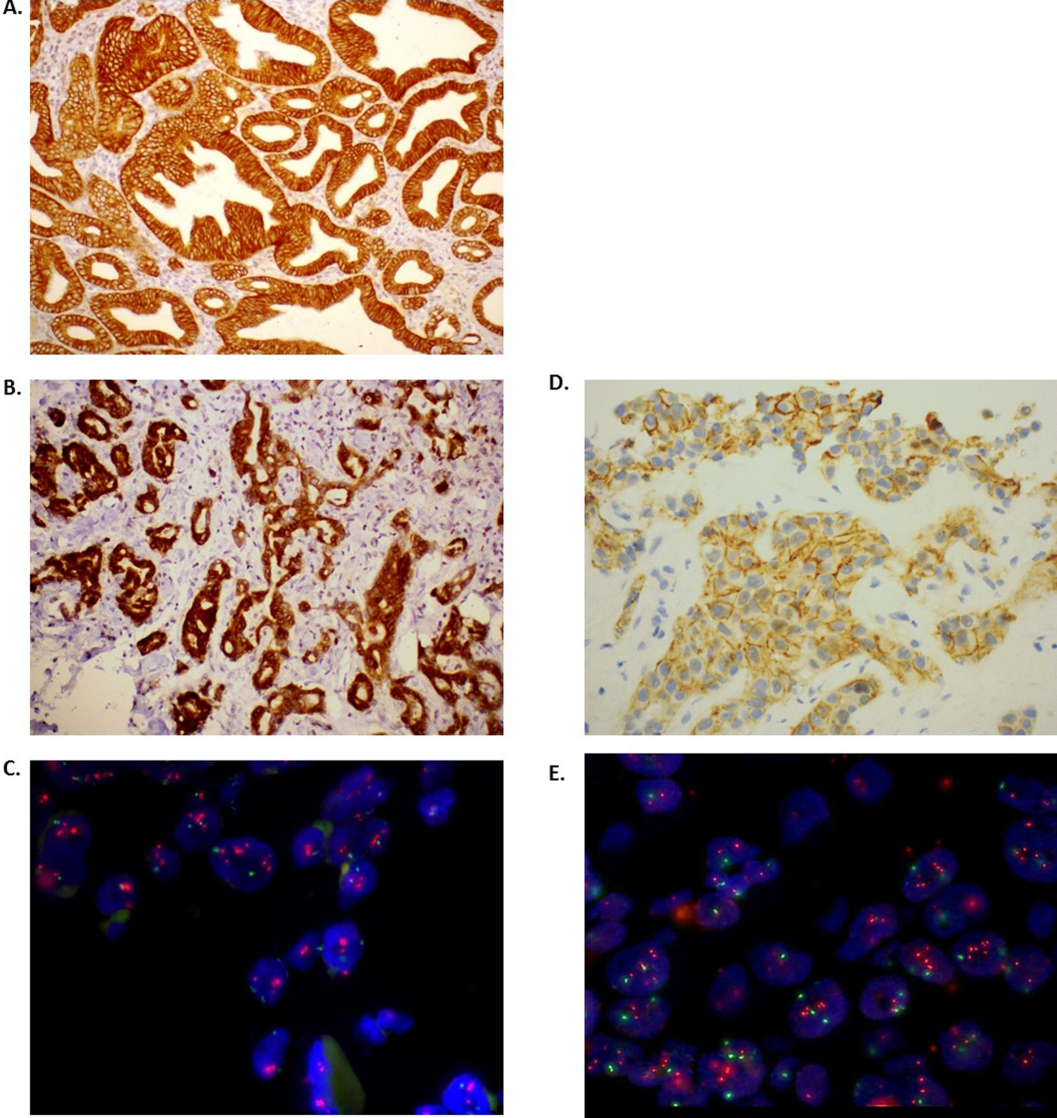
HER3 and HER2 expression in biliary tract cancer. Fig 1A. and Fig 1B. show immunohistochemistry (IHC) staining demonstrating HER3 protein expression (brown, 3+ intensity) in a background of blue Haematoxylin staining to illustrate the cellular outline, Fig 1A. indicates an example of HER3 membrane expression (brown) and Fig 1B. shows an example of HER3 cytoplasmic expression (brown). Fig 1C. Shows fluorescent in situ hybridisation (FISH) to demonstrate HER3 gene amplification. Fig 1D. shows immunohistochemistry (IHC) staining demonstrating HER2 protein membrane expression (brown, 2+ intensity) in a background of blue Haematoxylin staining to illustrate the cellular outline. Fig 1E. Shows fluorescent in situ hybridisation (FISH) to demonstrate HER2 gene amplification (Ratio 2.25). HER3; human epidermal growth factor receptor 3; HER2; human epidermal growth factor receptor 2.

Examples of HER2 and HER3 IHC and FISH staining are shown in Fig 1. S3 Table illustrates a heat-map summary of all the HER2 and HER3 protein expression (IHC) and gene amplification (FISH) from this study.

**Fig 2:**
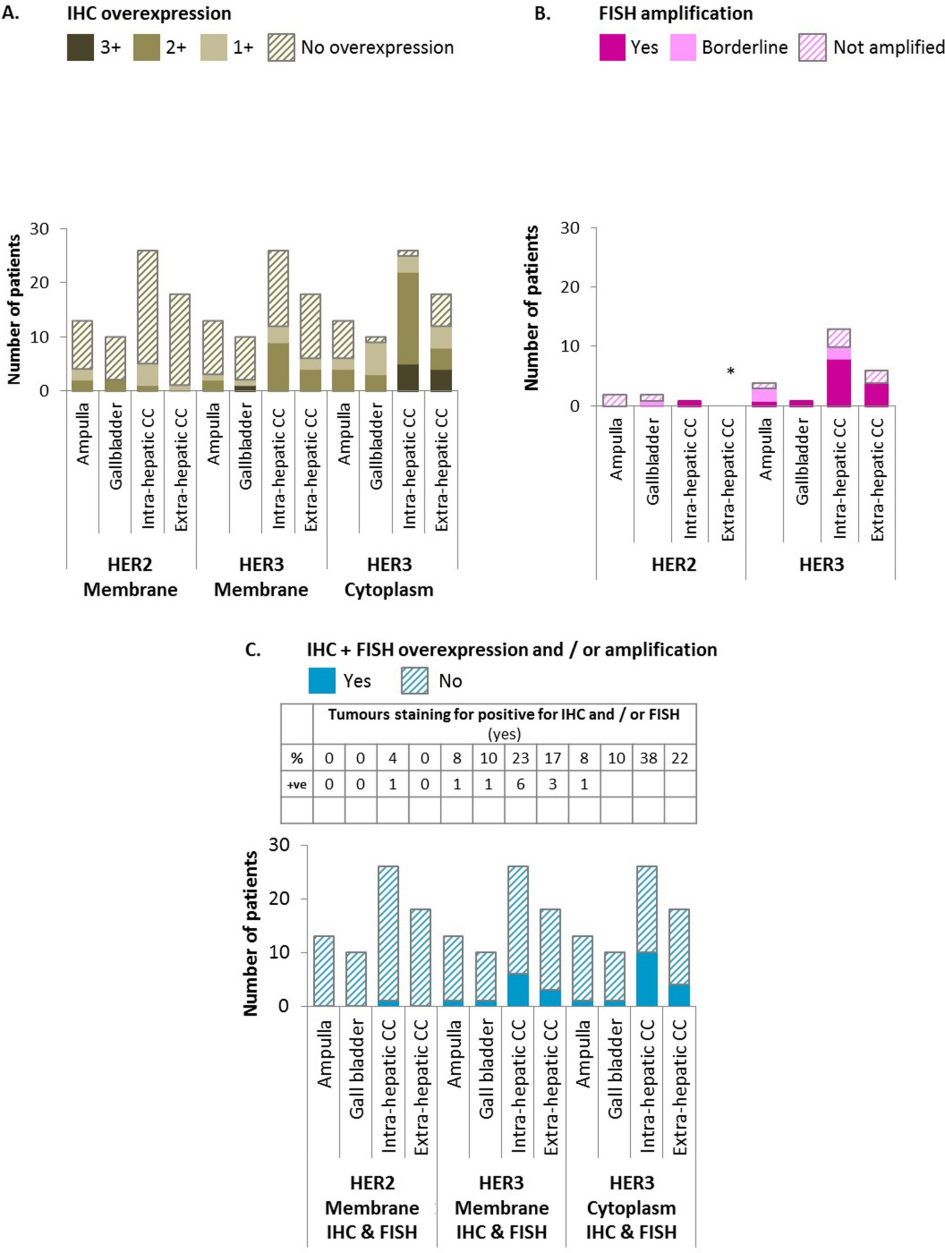
Summary of HER2 and HER3 expression and amplification in biliary tract cancer. Fig 2A. IHC staining demonstrated that the most prevalent staining observed in this study was HER3 cytoplasmic expression. Fig 2B. FISH staining demonstrated that of the patients tested, those with intra-hepatic CC had the most amplification of HER2 and HER3, * no extra-hepatic CC patients were eligible for HER2 FISH testing. Fig 2C. Considering the combination of IHC and FISH staining together, the most prevalent combined staining observed was HER3 cytoplasmic expression, and this was predominantly in patients with intra- and extra-hepatic CC. Tables provided in each figure summarise the results of each one of the scenarios explored. Percentages (%) are calculated for Fig 2A and 2C using the total number (Tot) of such subgroup in the whole series as a denominator (ampulla: 13 patients, gallbladder: 10 patients, intrahepatic cholangiocarcinoma: 26 patients, extrahepatic cholangiocarcinoma: 13 patients); for Fig 2B, the number of patients in each subgroup undergoing FISH analysis is used as a denominator instead (Tot) (ampulla: 2 patients, gallbladder: 2 patients, intrahepatic cholangiocarcinoma: 1 patient, extrahepatic cholangiocarcinoma: 0 patients). The row “+ve” represents the number of patients from each subgroup with positive results: for Fig 2A. IHC overexpression of 3+ is considered positive; for Fig 2B, presence of amplification in FISH (labelled as “yes”) is considered positive; for Fig 2C, 3+ in IHC and/or amplification in FISH is considered as positive. CC; cholangiocarcinoma, FISH; fluorescence in-situ hybridisation, HER2 and HER3; human epidermal growth factor receptors 2 and 3, IHC; immunohistochemistry.

HER2 overexpression by IHC (IHC3+) was not identified in any patients (Fig 2A). Of the five patients with HER2 IHC2+ who underwent FISH analysis (Fig 2B), HER2 amplification was confirmed in only one patient (a second patient was classified as “borderline”). Thus, in total, HER2 overexpression/amplification was only identified in 1.5% of the whole population (Fig 2C).

Based on IHC alone (Fig 2A), HER3 membrane overexpression (IHC2+) was identified in one patient with GBC only. Cytoplasmic overexpression was more frequent (identified in 9 patients: 5 ICC and 4 ECC). When FISH was performed (Fig 2B) on moderately staining (IHC2+) cases, 14/24 had HER3 expression classified as ‘positive’. When FISH results were analysed jointly with IHC (Fig 2C), rate of overexpression/amplification of HER3 in membrane and cytoplasm increased up to 16% [AMP (1 patient), GBC (1 patient), ICC (6 patients), ECC (3 patients)] and 24% [AMP (1 patient), GBC (1 patient), ICC (10 patients), ECC (4 patients)], respectively. There was a trend for a predominance of membrane/cytoplasmic HER3 overexpression/amplification in patients with ICC compared to others. However, logistic regression investigating factors predictive of expression of HER3 did not confirm such findings (S4 Table). Eleven patients presented with co-expression of both membrane and cytoplasmic HER3 overexpression/amplification, while six patients had cytoplasmic overexpression/amplification without membrane overexpression/amplification. None of the patients had membrane overexpression/amplification in the absence of cytoplasmic membrane overexpression/amplification.

HER2 and HER3 overexpression/amplification did not vary significantly between patients with early (stage I-II) and advanced (stage III-IV) disease (full data not shown).

Even though analysis of co-expression was limited due to the small number of patients with overexpression/amplification of HER2, only one patient showed co-expression of both HER2 and HER3. Factors predictive of HER2/3 overexpression.

Factors predicting HER2 overexpression/amplification could not be explored because data from only one patient was relevant. Factors predicting membrane/cytoplasmic HER3 overexpression/amplification are summarised in S4 Table. Male gender was the only factor related to increased risk of HER3 expression (Odds Ratio (OR) 4.65 (95% CI 1.31–16.45) in the univariate analysis. No multivariable analysis could be performed as only one factor was significant.

### Survival analysis and correlation with response to palliative chemotherapy

Most patients (82.09%) had died at the time of data analysis, after a median follow-up of 15.93 months. Estimated median OS for all patients was 15.9 months (95% confidence interval [CI] 11.1–20.3). Survival analysis is summarised in Table 2 (Column A for whole series; Column B for patients with localised stages; Column C for patients with advanced stages). Differing tumour sites reflected changes in patients’ outcomes; patients with ECC and ICC had an increased risk of death. Neither HER2 nor HER3 protein overexpression/amplification were prognostic. The impact of either membrane or cytoplasmic expression/amplification as a prognostic factor was assessed: neither impacted on OS (full data not shown).

**Table 2:** Multivariable Cox regression for Overall Survival.

Survival analysis		Column A (all patients; N = 67)		Column B (stage I-II disease; N = 21)		Column C (stage III-IV disease; N = 46)	
		Univariate Cox Regression (HR (95% CI); p-value)	Multivariable Cox Regression (HR (95% CI); p-value)	Univariate Cox Regression (HR (95% CI); p-value)	Multivariable Cox Regression (HR (95% CI); p-value)	Univariate Cox Regression (HR (95% CI); p-value)	Multivariable Cox Regression (HR (95% CI); p-value)
**Age (years)**	Cont variable	1.03 (1.00–1.06); 0.027	1.06 (1.02–1.09); 0.002	1.09 (1.02–1.17); 0.009	1.09 (1.01–1.18); 0.029	1.01 (0.98–1.04); 0.401	**-**
**Gender**	Male (vs Female)	0.72 (0.42–1.23); 0.227	-	1.16 (0.38–3.49); 0.797	-	0.61 (0.033–1.16); 0.132	-
**ECOG Performance status**	≥2 (vs 0–1)	0.93 (0.49–1.74); 0.816	-	0.28 (0.05–1.33); 0.110	-	2.45 (1.24–4.86); 0.010	4.27 (1.51–12.10); 0.006
**Primary site**	ICC	1 (Ref)	1 (Ref)	1 (Ref)	-	1 (Ref)	1 (Ref)
	ECC	0.49 (0.26–0.96); 0.037	0.45 (0.21–0.98); 0.043	0.63 (0.12–3.27); 0.582	-	0.89 (0.41–1.98); 0.792	0.49 (0.19–1.29); 0.152
	Ampullary cancer	0.15 (0.06–0.38); <0.001	0.05 (0.01–0.17); <0.001	0.35 (0.62–2.01); 0.241		0.05 (0.01–0.41); 0.005	3.64x10^-16^ (cannot calculate)
	Gallbladder	0.79 (0.36–1.70); 0.535	1.28 (0.57–3.03); 0.568	0.36 (0.03–4.18); 0.412	-	1.36 (0.59–3.11); 0.459	0.98 (0.62–3.03); 0.968
**Primary site**	ECC (vs ICC)	0.49 (0.26–0.96); 0.039	x	0.59 (0.11–3.31); 0.554	-	0.89 (0.40–1.95); 0.764	x
**Tumour differentiation**	Poorly (vs Well/Mod)	1.27 (0.67–2.38); 0.465	-	1.08 (0.33–3.53); 0.905	-	5.94 (2.34–15.07); <0.001	2.95 (1.03–8.42); 0.043
**Stage**	III-IV (vs. I-II)	3.2 (1.69–6.06); <0.001	4.37 (1.56–7.75); 0.002	n/a	n/a	n/a	n/a
**Ca19.9**	Cont variable	1.00 (1.001–1.002); <0.001	1.00 (1.001–1.01); 0.046	1.001 (1.0001–1.01); 0.031	1.001 (0.99–1.01); 0.491	1.001 (1.0001–1.01); 0.001	1.001 (1.0001–1.01); 0.008
**Albumin**	Cont variable	0.90 (0.82–0.99); 0.028	0.83 (0.74–0.92); 0.001	0.81 (0.63–1.03); 0.086	-	0.94 (0.85–1.04); 0.250	-
**NLR**	Cont variable	1.09 (1.04–1.16); <0.001	1.03 (0.96–1.10); 0.385	1.81 (1.12–2.94); 0.016	1.40 (0.87–2.27); 0.168	1.05 (0.99–1.12); 0.066	-
**Her 2 membrane overexpression / amplification**	Yes (vs No)	5.48 (0.71–42.48); 0.103	-	Cannot be calculated	-	3.57 (0.46–27.63); 0.224	-
**Her 3 membrane/cytoplasmic overexpression / amplification**	Yes (vs No)	1.57 (0.86–2.85); 0.138	-	1.35 (0.41–4.38); 0.622	-	2.14 (1.04–4.42); 0.039	1.02 (0.41–2.56); 0.963
**Her 3 membrane overexpression / amplification**	Yes (vs No)	1.37 (0.67–2.81); 0.391	-	0.95 (0.21–4.37); 0.950	-	1.79 (0.78–4.13); 0.168	-
**Her 3 cytoplasmic overexpression / amplification**	Yes (vs No)	1.57 (0.86–2.85); 0.138 (&)	-	1.35 (0.41–4.38); 0.622 (&)	-	2.14 (1.04–4.42); 0.039 (&)	&

ECOG: Eastern Cooperative Oncology Group; 95%CI: 95% confidence interval; CA19.9: cancer antigen 19–9; CC: cholangiocarcinoma; HR: hazard ratio; NLR: neutrophil/lymphocyte ratio; Ref: reference category; N: number of patients; n/a: not applicable; Cont: continuous.

#: included in the multivariable analysis in the form of NLR.

&: because there were no patients with membrane expression in the absence of cytoplasmic expression, the behaviour of this variable mimics “Her 3 membrane/cytoplasmic overexpression / amplification” in the survival analysis, including multivariable analysis.

Median PFS in patients with advanced BTC receiving first-line palliative chemotherapy was 7.7 months (95% CI 4.24–8.60). PFS was similar for HER3 positive/negative patients (log-rank test p-value 0.5745 for patients treated with gemcitabine and platinum; log-rank test p-value0.4632 for patients treated with single agent gemcitabine). A total of 24.2% of patients achieved a partial response (16.67% of patients in gemcitabine single agent, 25.95% treated with gemcitabine and platinum). Response rate did not vary between HER3 positive/negative patients (logistic regression p-value 1 for patients treated with single agent gemcitabine; logistic regression p-value 0.852 for patients treated with gemcitabine and platinum).

### CbioPortal “in silico” analysis

**Fig 3:**
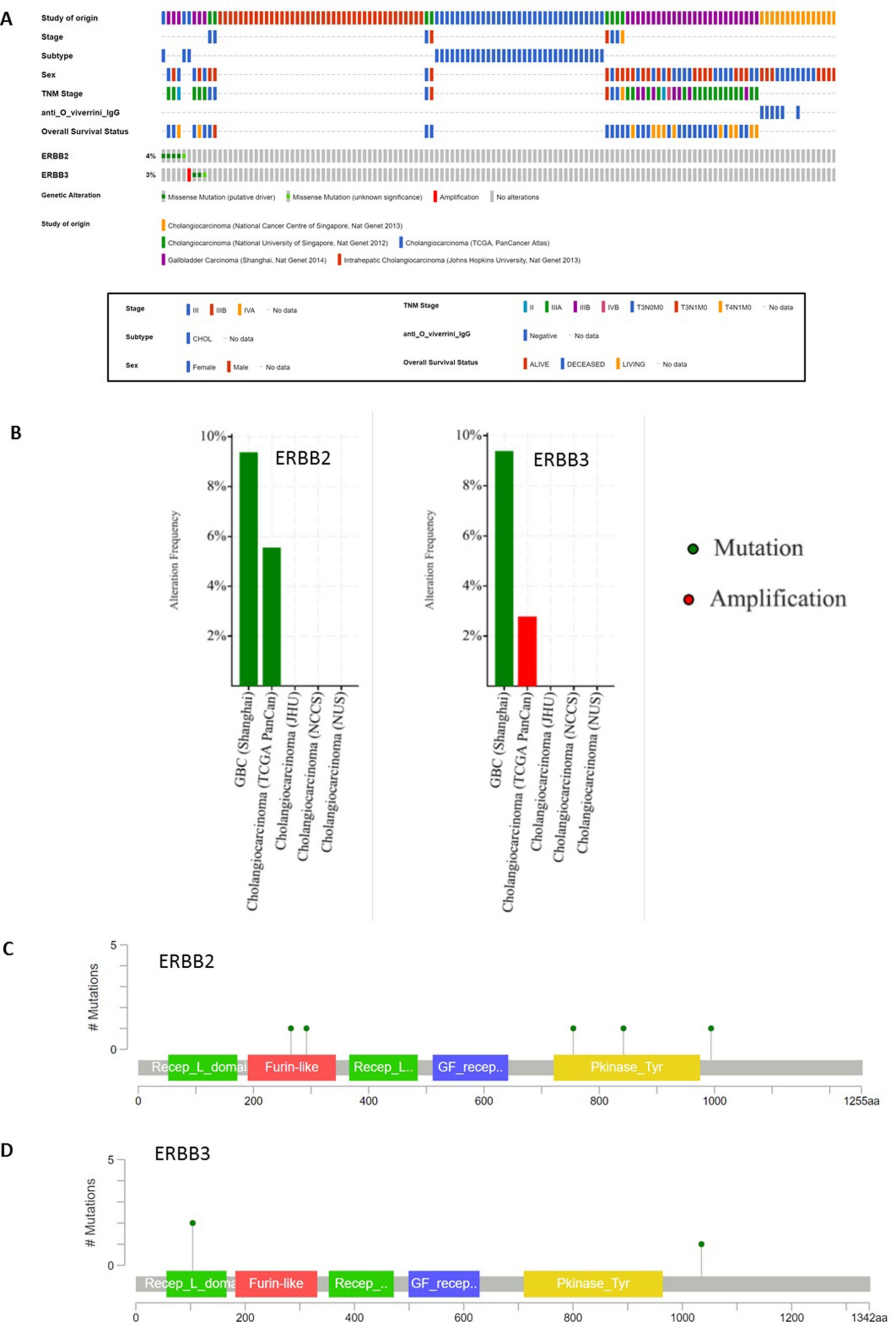
CbioPortal “in silico” analysis of data available from the genomic consortia (131 patients are included). Fig 3A ERBB (HER) 2 & 3 analysis of CbioPortal data. Fig 3B summary of data by dataset. Fig 3C “lollipop” diagrams for ERBB 2 (green lines represent missense mutations). Fig 3D “lollipop” diagrams for ERBB 3 (green lines represent missense mutations).

Data from CbioPortal show that mutations in HER2 and HER3 are infrequent (4% and 3% respectively (Fig 3A) and mutually exclusive (Fig 3A); amplification of HER2 was not identified, while 1 patient (diagnosed with moderately differentiated cholangiocarcinoma) had HER3 amplified (Fig 3A and 3B)). HER2 and HER3 mutations were widely spread through the whole exome of both genes (Fig 3C and 3D); two of the HER2 mutations are located in the kinase domain (one patient with intrahepatic cholangiocarcinoma and a second patient with gallbladder cancer) (Fig 3C); none of the HER3 mutations were located in kinase domain; two were located in the receptor L domain (site of ligand binding) (Fig 3D).

## Discussion

New treatment targets are needed for patients with BTC [5]. HER3 is becoming an attractive target in cancer for selected patients [32,33]. Unfortunately, studies exploring the prevalence of HER2 and HER3 expression in BTC have been of variable quality, not using standardised criteria and focused mainly on HER2 expression; HER3 expression has been underexplored [26].

One of the main challenges for assessment of HER2 and HER3 is the lack of standardised criteria [34]. Following comparison between breast [29] and gastric [27,28] criteria for HER2 assessment in this study, the gastric criteria was found to be most appropriate because of similar tumour morphology. In addition, this strategy avoids underestimating HER2 expression, which was apparent when the breast criteria were used in BTC. Thus, the gastric criteria are recommended in future studies exploring HER2 or HER3 in BTC using IHC or FISH. It is also recommended that FISH is only performed on patients with IHC2+ staining-tumours. The addition of FISH to all patients assessed with IHC is not supported by current guidelines [29,27,28] or other large series [35].

The current results have identified HER3 as a potential target which warrants further investigation in BTC, as it was expressed in a significant subset of patients diagnosed with BTC. Previously, HER3 expression has been examined in several studies including BTC patients, and are summarised in Table 3[36,37,20,38,26]. HER3 expression varies between studies, due to the use of different scoring systems and differing definitions of “expression” [36,20]. In addition, in some studies the cytoplasmic expression is not separately reported [38], thus, confounding the results and making comparisons difficult. When the current series is compared with studies with similar methodology, results on HER3 expression are similar [37]. Data from CbioPortal supports that HER3 amplification is present in BTCs (Fig 3). Because mutations identified in HER3 are usually not located in the kinase domain, it may be that overexpression/amplification rather than mutation could be targeted in BTC using an antibody approach [32,34,25,39].

**Table 3:** Studies exploring HER2 and HER3 expression by immunohistochemistry (IHC) and/or amplification by *in situ* hybridisation (ISH) in biliary tract carcinomas.

Study	Country	N	Primary	IHC scoring / type sample	Internationally agreed criteria for IHC assessment? / HER2 membrane expression reported separately?/ HER3 membrane expression reported separately?	HER2 IHC		HER2 FISH		HER3 IHC		HER3 FISH		HER2/HER3 co-expression
						N	%*	N	%*	N	%*	N	%*	
**Lee HJ 2012[36]**	South Korea (Asian)	230	ECC	staining intensity and percentage of positive cells; HER2 expression was located in membrane while HER3 was located in cytoplasm / TMA	No/No/No	13/224	6%	NR	NR	90/230	39%	NR		7/230 (3%)
**Yang X 2014[37]**	China (Asian)	65	ICC	Classified as negative/weak/moderate/strong ^[^^60^^]^ / TMA	No/No/No	8/65	12.3%	8/65	12.3%	8/65	12.3%	NR		NR
		110	ECC			13/110	11.8%	13/110	11.8%	13/110	11.8%	NR		
**Kawamoto T 2015[20]**	USA	47	GBC	A four-point scale: 0, 1+, 2+, and 3+; score of 2+ was considered overexpression / whole slides	No/Yes/Yes	15/47	32%	8/47	17%	16/47	34%	12/47	26%	NR
	Japan (Asian)	66	CC			15/66	23%	15/66	23%	19/66	29%	18/66	27%	
**Yan et al 2015[35]**	USA	194	GBC	As per ASCO guidelines; whole slides	Yes/Yes/n/a	19/194	9.8%	NR		NR		NR		NR
		321	ICC			2/321	0.6%							
		80	ECC			5/80	6.3%							
**Galdy et al 2015 [26]**	17 studies (Asia) 16 studies (Western)	3839 patients	ICC 924; ECC 920; GBC 1026; AMP 303	Systematic review and meta-analysis	No/No/No	38 studies; Expression rate mean 26.5% (95% CI 18.9–34.1)		12 studies; Amplification rate mean 17.9% (95% CI 0.1–35.4)^&^		4 studies; Expression rate mean 27.9% (95% CI 9.7–46.1)		1 studies; Amplification rate 26.5% (95% CI n/a)		No correlation identified between HER2 and HER expression
**Elebro J 2016 [38]**	Sweden (Western)	175	AMP (all)	A four-point scale: 0, 1+, 2+, and 3+; 3+ was considered high expression / TMA	Yes/Yes/No	4/175	2%	3/175	3%	50/175	29%	NR		0/175 (0%)
		110	AMP (pancreato-biliary type)			0/110	0%	1/110	7%	18/110	17%			
		65	AMP (intestinal type)			4/65	6%	2/65	15%	32/65	51%			
**Lamarca et al 2018 (this series)**	UK (Western)	26	ICC	A four-point scale: 0, 1+, 2+, and 3+ (3+ was considered high expression / whole slides	Yes/Yes/Yes	0/26	0%	1/26	4%	0/26	0%	6/26	23%	1/67 (1%)
		18	ECC			0/18	0%	0/18	0%	0/18	0%	3/18	22%	
		13	AMP			0/13	0%	0/13	0%	0/13	0%	1/13	8%	
		10	GBC			0/10	0%	0/10	0%	1/10	10%	1/10	10%	

NR: not reported: ICC: intra-hepatic cholangiocarcinoma, ECC: extra-hepatic cholangiocarcinoma; AMP ampullary cancer; GBC: gallbladder cancer; 95% CI: 95% confidence interval; N: number of patients; %: percentage; IHC: immunohistochemistry; FISH: Fluorescent in situ hybridisation; HER: Human epidermal growth factor receptor; TMA: tissue micro-array; CC: cholangiocarcinoma; NR: not reported; n/a: not applicable.

* all percentages are calculated using the total number of samples included in the series as a denominator (the number of samples tested for FISH has not been used as a denominator due to discrepancy between studies (some studies performed FISH for every sample regardless of IHC results[37] [20], some studies performed FISH only if IHC2+/3+ [38] or 2+ (Lamarca et al, this series).

& there was variability regarding patients tested with FISH (selected based on IHC/non-selected), data for “un-selected” population as defined by authors are provided in the table [26].

However, the rate of HER2 expression in this study was below the reported rate to date, particularly when compared with other selected series [20,37], and data from previous meta-analysis [26] (Table 3). This is not an isolated discrepancy and has also been highlighted by other research teams [40].

There may be many reasons for this discrepancy. Firstly, geography could play a role and cannot be completely excluded[40,41]. A recent meta-analysis identified differences (although not statistically significant) in HER2 expression between Western (mean 19.7% (95% CI 10.1–29.2)) and Asian (mean 28.4% (95% CI 14.5–42.3)) populations [26]. The link between aberrations in the HER pathway especially in liver-fluke related BTC (more frequent in Asian countries) could also support this hypothesis [42]. Secondly, there is a clear discrepancy and inaccuracy in staining overexpression criteria [43,40,44], which are summarised in Table 3. Non-membrane location (cytoplasmic/nucleus) has sometimes been taken into account for the definition of HER expression and could explain such differences [34,45]. It is notable that similar HER2 expression (<10%) to our study was identified in one of the largest pan-cancer HER2 studies to date using high quality and internationally agreed IHC/FISH criteria [35]. Thirdly, tumour pathological and morphological characteristics may play a role, as suggested by Elebro and colleagues, who reported a significant variation in HER2 and HER3 expression according to AMP subtype (intestinal type (HER2 6%, HER3 51%) vs pancreato-biliary (HER2 0%, HER3 17%) type) [38].

When all these challenges are taken into account, it is likely that actual HER2 expression has been overestimated in previous series. This is also supported by data from CbioPortal, where no amplification of HER2 were identified (Fig 3) [31]. This suggests that mutations located in the HER2 kinase domain rather than amplification may be worth targeting in BTC [25]. Inadequate patient selection may explain previous negative trials with HER2-inhibitors in patients with BTC [5].

Co-expression of HER2 and HER3 is of interest, especially due to the close relationship between these two receptors [32]. This study showed co-expression of HER2 and HER3 in one patient only (1% of the whole population) which is in-keeping with other BTC series [38,36] and data from CbioPortal (Fig 3) [31]. Interestingly, while co-expression of HER2 and HER3 has been identified rarely in BTC [38,36] (Table 3), on the contrary this seems to be common in other [46,47]. [30] [48] This evidence could encourage targeting of HER3 in isolation in BTC, without the need of targeting HER2 concomitantly.

Studies in a variety of cancers suggest a link between HER3 expression and more advanced tumours [49] and also with worse outcome [50,51,52,34,30,53]. This study did not confirm HER3 to impact on patient outcomes or response to chemotherapy, as has also been suggested by other BTC series [54,36]. Thus, the prognostic role of HER3 in BTC remains unclear with conflicting results [55,38].

Similarly, no factors were identified that were related with increased risk of HER3 expression, as previously suggested (i.e. Yang and colleagues suggested a link between HER3 expression and poorly-differentiated BTC morphology [56]). This statement applies to primary site of BTC also. Thus, HER3 is worth exploring in the whole BTC spectrum.

Another important observation was the presence of HER3 in the cytoplasm of BTC cells. HER3 is usually localised at the cell membrane but is able to move into the nucleus and promote carcinogenesis. Interestingly, nuclear localisation of HER3 does not necessarily imply unfavourable cancer characteristics [34]. Currently, the presence of cytoplasmic HER3 is not completely understood and its biological implications remain unknown. The possibility of such expression being a reflection of artefact cannot be completely excluded. However, other series describe the same HER3 distribution [38,45,36,48] and so makes this unlikely. The underlying mechanism for nuclear localisation of HER3 remains unknown, and its presence is very dynamic due to its compensatory role in HER pathway signalling [34]. The cytoplasmic expression could be a reflection of intracellular trafficking. None of the patients in the current series had membrane expression in the absence of cytoplasmic expression, which could support this hypothesis.

Our results support HER3 as a potential treatment target in BTC. HER3 has a low-activity kinase domain, thus requires heterodymerisation with other HER family members (HER1/HER2) for efficient signalling [32]. Even though inhibition of HER3 has been under-explored for years, the interest on drugs targeting HER3 has recently increased significantly [33,34,57]. HER3 inhibition may be achieved through different strategies such as, but not limited to, the inhibition through direct antibody bindings, inhibition of dimerization, Inhibition of tyrosine kinase activity or Inhibition of ligands. Some of the strengths of this work can be summarised as follows: First, the current series had representation of all BTC spectrum of malignancies and outcomes were in-keeping with previous experience (i.e. both OS, PFS were in keeping with previous studies [58] and ampullary malignancies were found to have the best outcomes [59]); thus is representative of real world data. Secondly, the study was performed using a pre-planned sample size calculation to ensure that it had sufficient power to identify clinically meaningful HER2/HER3 overexpression/amplification. This allowed for conclusions to be robust and clinically meaningful (based on pre-defined clinical parameters). From this sample size calculation, expression of 5% would be defined as of “no interest”, while expression of 15% or above would be defined as “worth exploring further”. Based on such pre-defined thresholds, it was concluded that while HER2 may not be of interest in BTC, HER3 should be explored further, not only from a mechanistic perspective, but also from a cause-effect and therapeutic point of view.

In summary, this study concluded that based on standardised high quality assessment of HER3 using IHC and FISH, HER3 is a target worth considering for future research in BTC using. Gastric IHC criteria is recommended for future IHC assessment in BTC exploring HER2 and HER3. The field would benefit from mechanistic studies exploring the real impact of HER3 overexpression/amplification in BTC cells, to understand the clinical significance of such findings and the role of targeting such proteins. Since the biological significance of HER3 cytoplasmic expression is not fully understood, whether patients with membrane HER3 expression only (excluding cytoplasmic expression) should be targeted, warrants further investigation.

## Supporting information

S1 Table Assessment of HER2 expression and amplification using gastric guidelines [28,27] and breast criteria [29]. CEP17; chromosome enumeration probe 17, HER2; human epidermal growth factor receptor 2.
(DOC)

S1 Fig The patient selection process for this study. Patients were recruited from January 2013 to July 2015.
(TIF)

S2 Table Number of patients classified with each one of the criteria (breast/gastric) and agreement between both criteria (grey cells) is shown. IHC: immunohistochemistry.
(DOC)

S3 Table Summary of HER2 and HER3 expression (IHC) and amplification (FISH). CC; cholangiocarcinoma, FISH; fluorescence in-situ hybridisation, HER2; human epidermal growth factor receptor 2, IHC; immunohistochemistry, n/a; not applicable.
(DOC)

S4 Table Factors predictive of HER3 membrane/cytoplasmic overexpression/amplification. OR: Odds ratio, 95% CI: 95% confidence interval; ECOG: Eastern Cooperative Oncology Group; ICC: intrahepatic cholangiocarcinoma; ECC: extrahepatic cholangiocarcinoma; NLR: neutrophil-lymphocyte ratio.
(DOC)
