# Correction: The HER3 pathway as a potential target for inhibition in patients with biliary tract cancers

**DOI:** 10.1371/journal.pone.0212697

**Published:** 2019-02-15

**Authors:** The PLOS ONE Staff

Fig 2 is incorrect. Please see the corrected Fig 2 here. The publisher apologizes for the error.

**Fig 2:**
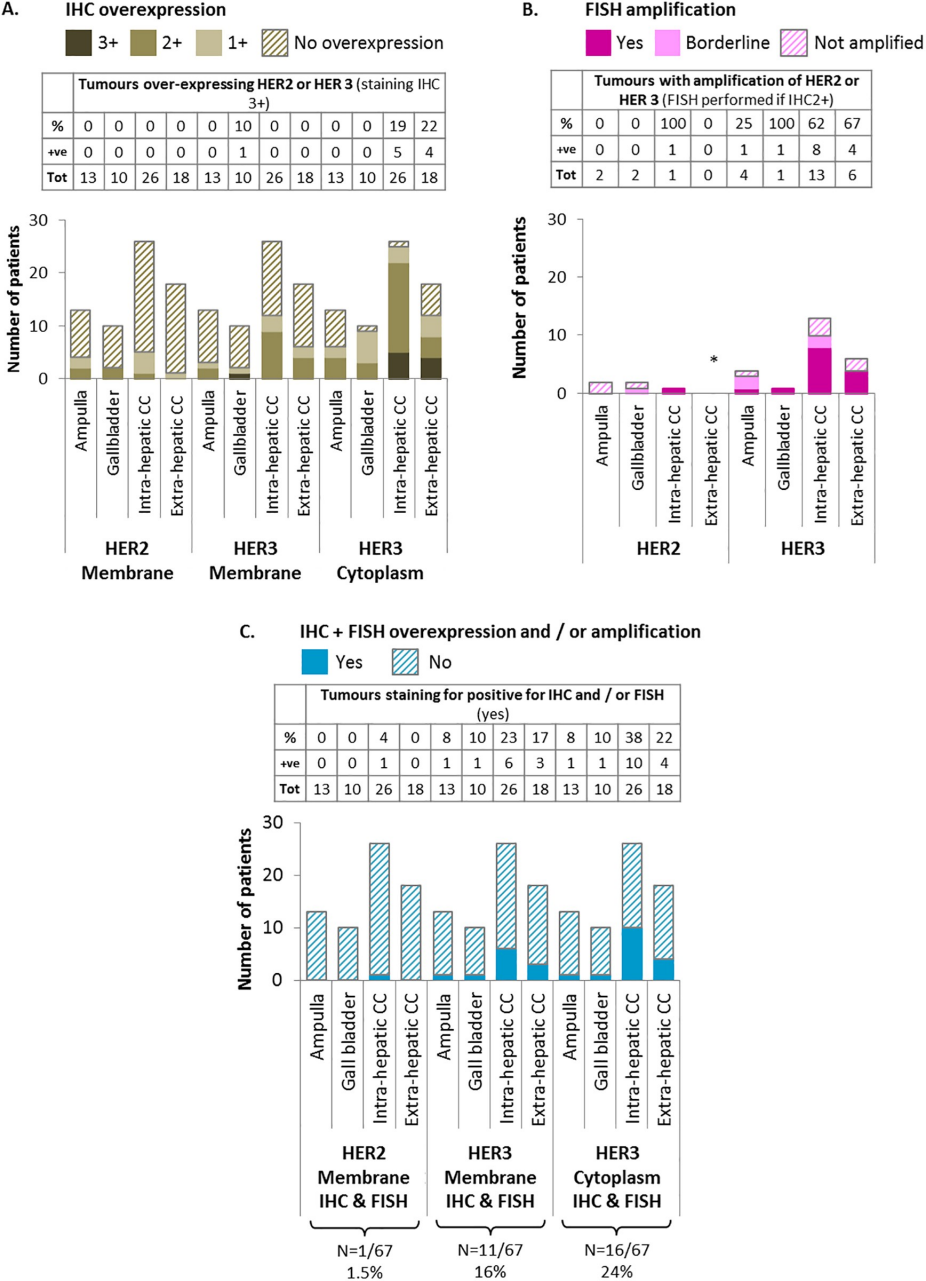
Summary of HER2 and HER3 expression and amplification in biliary tract cancer. Fig 2A. IHC staining demonstrated that the most prevalent staining observed in this study was HER3 cytoplasmic expression. Fig 2B. FISH staining demonstrated that of the patients tested, those with intra-hepatic CC had the most amplification of HER2 and HER3, * no extra-hepatic CC patients were eligible for HER2 FISH testing. Fig 2C. Considering the combination of IHC and FISH staining together, the most prevalent combined staining observed was HER3 cytoplasmic expression, and this was predominantly in patients with intra- and extra-hepatic CC. Tables provided in each figure summarise the results of each one of the scenarios explored. Percentages (%) are calculated for Fig 2A and 2C using the total number (Tot) of such subgroup in the whole series as a denominator (ampulla: 13 patients, gallbladder: 10 patients, intrahepatic cholangiocarcinoma: 26 patients, extrahepatic cholangiocarcinoma: 13 patients); for Fig 2B, the number of patients in each subgroup undergoing FISH analysis is used as a denominator instead (Tot) (ampulla: 2 patients, gallbladder: 2 patients, intrahepatic cholangiocarcinoma: 1 patient, extrahepatic cholangiocarcinoma: 0 patients). The row “+ve” represents the number of patients from each subgroup with positive results: for Fig 2A. IHC overexpression of 3+ is considered positive; for Fig 2B, presence of amplification in FISH (labelled as “yes”) is considered positive; for Fig 2C, 3+ in IHC and/or amplification in FISH is considered as positive. CC; cholangiocarcinoma, FISH; fluorescence in-situ hybridisation, HER2 and HER3; human epidermal growth factor receptors 2 and 3, IHC; immunohistochemistry.
