# Supplementary material for: The HER3 pathway as a potential target for inhibition in patients with biliary tract cancers
Source: PLoS One. 2018 Oct 18;13(10):e0206007. doi: 10.1371/journal.pone.0206007 (PMC6193702; doi:10.1371/journal.pone.0206007)
Supplement: S1 Table — CEP17; chromosome enumeration probe 17, HER2; human epidermal growth factor receptor 2. (DOC) [file pone.0206007.s001.doc]

## S1 Table. Assessment of HER2 expression and amplification using gastric guidelines [28,27] and breast criteria [29].

CEP17; chromosome enumeration probe 17, HER2; human epidermal growth factor receptor 2.

|  | **Staining** | **Gastric cancer** ^[28,27]^ | **Breast cancer** ^[29]^ |
| --- | --- | --- | --- |
| **HER2 expression** (IHC) | **0+** (negative) | No reactivity or membranous reactivity in <10% of cells  **Biopsy sample:** No reactivity | No staining. Incomplete membrane faint/barely perceptible staining within  ≤ 10% of the invasive tumour cells |
|  | **1+**  (weak; negative) | Faint⁄barely perceptible partial membranous reactivity in >10% of cells  **Biopsy sample:** Faint⁄barely reactive | Incomplete membrane faint/barely perceptible staining within >10% of the invasive tumour cells |
|  | **2+** (moderate; equivocal) | Weak/moderate complete or basolateral membranous reactivity in >10% of tumour cells  **Biopsy sample:** Weak/moderate reactivity | Circumferential, weak/moderate and/or incomplete membrane staining within >10 % of the cancerous cells. Complete circumferential intense membrane within ≤ 10% of the invasive tumour cells |
|  | **3+**  (strong; positive) | Moderate/strong complete or basolateral membranous reactivity in >10% of tumour cells  **Biopsy sample:** Strong reactivity | Circumferential, intense complete membrane staining in >10 % of the cancerous cells |
| **HER2 amplification** (FISH) | | **FISH amplified positive:** IHC/HER2 2+ tumour samples are considered FISH amplified if HER2:CEP17 ratio is ≥2 | **HER2 FISH testing positive:** (gene copy number and HER2-to-CEP17 ratio) HER2 gene copy number is greater than 6.0 (single probe) and in case of HER2 2+, if either HER2:CEP17 ratio is ≥2.0 regardless of gene copy number or if HER2:CEP17 ratio is <2.0 with an average HER2 copy number ≥6.0 (dual probe) |

CEP17; chromosome enumeration probe 17, HER2; human epidermal growth factor receptor 2.
