# Supplementary figures and images for: The HER3 pathway as a potential target for inhibition in patients with biliary tract cancers
Source: PLoS One. 2018 Oct 18;13(10):e0206007. doi: 10.1371/journal.pone.0206007 (PMC6193702; doi:10.1371/journal.pone.0206007)

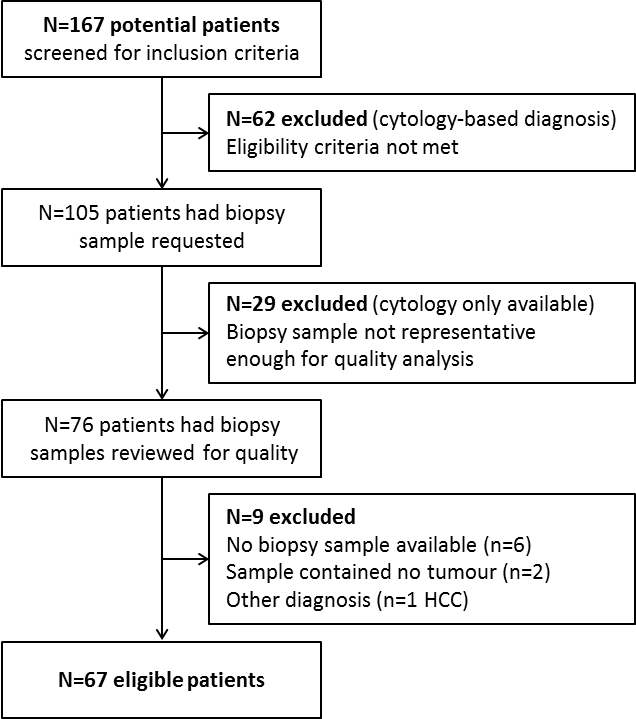

Supplement: S1 Fig — Patients were recruited from January 2013 to July 2015. (TIF) [file pone.0206007.s002.tif]
