# Supplementary material for: The HER3 pathway as a potential target for inhibition in patients with biliary tract cancers
Source: PLoS One. 2018 Oct 18;13(10):e0206007. doi: 10.1371/journal.pone.0206007 (PMC6193702; doi:10.1371/journal.pone.0206007)
Supplement: S2 Table — IHC: immunohistochemistry. (DOC) [file pone.0206007.s003.doc]

## S2 Table. Number of patients classified with each one of the criteria (breast/gastric) and agreement between both criteria (grey cells) is shown.

|  | | **Gastric IHC criteria** | | | |
| --- | --- | --- | --- | --- | --- |
|  |  | **IHC 0** | **IHC 1+** | **IHC 2+** | **IHC 3+** |
| **Breast IHC criteria** | **IHC 0** | 20 | 5 | 0 | 0 |
|  | **IHC 1+** | 0 | 0 | 1 | 0 |
|  | **IHC 2+** | 0 | 0 | 1 | 0 |
|  | **IHC 3+** | 0 | 0 | 0 | 0 |

IHC: immunohistochemistry
