# Supplementary material for: The HER3 pathway as a potential target for inhibition in patients with biliary tract cancers
Source: PLoS One. 2018 Oct 18;13(10):e0206007. doi: 10.1371/journal.pone.0206007 (PMC6193702; doi:10.1371/journal.pone.0206007)
Supplement: S3 Table — CC; cholangiocarcinoma, FISH; fluorescence in-situ hybridisation, HER2; human epidermal growth factor receptor 2, IHC; immunohistochemistry, n/a; not applicable. (DOC) [file pone.0206007.s004.doc]

## S3 Table. Summary of HER2 and HER3 expression (IHC) and amplification (FISH).

CC; cholangiocarcinoma, FISH; fluorescence in-situ hybridisation, HER2; human epidermal growth factor receptor 2, IHC; immunohistochemistry, n/a; not applicable.

|  |  |  | **All patients** | | **Ampullary** | | **Gallbladder** | | **Intra-hepatic CC** | | **Extra-hepatic CC** | |
| --- | --- | --- | --- | --- | --- | --- | --- | --- | --- | --- | --- | --- |
| **Target** | **Staining** | **Intensity** | **N** | **%** | **N** | **%** | **N** | **%** | **N** | **%** | **N** | **%** |
| **HER2** | **IHC staining membrane** | 0 | 55/67 | 82 | 9/13 | 69 | 8/10 | 80 | 21/26 | 81 | 17/18 | 94 |
|  |  | 1+ | 7/67 | 10 | 2/13 | 15 | 0/10 | 0 | 4/26 | 18 | 1/18 | 6 |
|  |  | 2+ | 5/67 | 7 | 2/13 | 15 | 2/10 | 20 | 1/26 | 4 | 0/18 | 0 |
|  |  | 3+ | 0/67 | 0 | 0/13 | 0 | 0/10 | 0 | 0/26 | 0 | 0/18 | 0 |
|  | **FISH** | No | 3/5 | 60 | 2/2 | 100 | 1/2 | 50 | 0/1 | 0 | n/a | n/a |
|  |  | Borderline | 1/5 | 20 | 0/2 | 0 | 1/2 | 50 | 0/1 | 0 | n/a | n/a |
|  |  | Yes | 1/5 | 20 | 0/2 | 0 | 0/2 | 0 | 1/1 | 100 | n/a | n/a |
|  | **Membrane IHC + FISH** | No | 66/67 | 99 | 13/13 | 100 | 10/10 | 100 | 25/26 | 96 | 18/18 | 100 |
|  |  | Yes | 1/67 | 1 | 0/13 | 0 | 0/10 | 0 | 1/26 | 4 | 0/18 | 0 |
| **HER3** | **IHC staining membrane** | 0 | 44/67 | 66 | 10/13 | 77 | 8/10 | 80 | 14/26 | 54 | 12/18 | 67 |
|  |  | 1+ | 7/67 | 10 | 1/13 | 8 | 1/10 | 10 | 3/26 | 12 | 2/18 | 11 |
|  |  | 2+ | 15/67 | 22 | 2/13 | 15 | 0/10 | 0 | 9/26 | 35 | 4/18 | 22 |
|  |  | 3+ | 1/67 | 1 | 0/13 | 0 | 1/10 | 10 | 0/26 | 0 | 0/18 | 0 |
|  | **IHC staining cytoplasm** | 0 | 15/67 | 22 | 7/13 | 54 | 1/10 | 10 | 1/26 | 4 | 6/18 | 33 |
|  |  | 1+ | 15/67 | 22 | 2/13 | 15 | 6/10 | 60 | 3/26 | 12 | 4/18 | 22 |
|  |  | 2+ | 28/67 | 42 | 4/13 | 31 | 3/10 | 30 | 17/26 | 65 | 4/18 | 22 |
|  |  | 3+ | 9/67 | 13 | 0/13 | 0 | 0/10 | 0 | 5/26 | 19 | 4/18 | 22 |
|  | **FISH** | No | 6/24 | 25 | 1/4 | 25 | 0/1 | 0 | 3/13 | 23 | 2/6 | 33 |
|  |  | Borderline | 4/24 | 17 | 2/4 | 50 | 0/1 | 0 | 2/13 | 15 | 0/6 | 0 |
|  |  | Yes | 14/24 | 58 | 1/4 | 25 | 1/1 | 100 | 8/13 | 62 | 4/6 | 67 |
|  | **Membrane IHC + FISH** | No | 56/67 | 84 | 12/13 | 92 | 9/10 | 90 | 20/26 | 77 | 15/18 | 83 |
|  |  | Yes | 11/67 | 16 | 1/13 | 8 | 1/10 | 10 | 6/26 | 23 | 3/18 | 17 |
|  | **Cytoplasm IHC + FISH** | No | 51/67 | 76 | 12/13 | 92 | 9/10 | 90 | 16/26 | 62 | 14/18 | 78 |
|  |  | Yes | 16/67 | 24 | 1/13 | 8 | 1/10 | 10 | 10/26 | 38 | 4/18 | 22 |


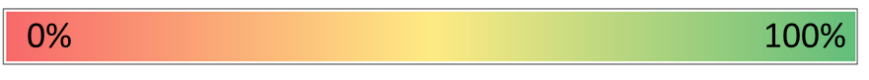


CC; cholangiocarcinoma, FISH; fluorescence in-situ hybridisation, HER2; human epidermal growth factor receptor 2, IHC; immunohistochemistry, n/a; not applicable.
