# Supplementary material for: The HER3 pathway as a potential target for inhibition in patients with biliary tract cancers
Source: PLoS One. 2018 Oct 18;13(10):e0206007. doi: 10.1371/journal.pone.0206007 (PMC6193702; doi:10.1371/journal.pone.0206007)
Supplement: S4 Table — OR: Odds ratio, 95% CI: 95% confidence interval; ECOG: Eastern Cooperative Oncology Group; ICC: intrahepatic cholangiocarcinoma; ECC: extrahepatic cholangiocarcinoma; NLR: neutrophil-lymphocyte ratio. (DOC) [file pone.0206007.s005.doc]

## S4 Table: Factors predictive of HER3 membrane/cytoplasmic overexpression/amplification.

OR: Odds ratio, 95% CI: 95% confidence interval; ECOG: Eastern Cooperative Oncology Group; ICC: intrahepatic cholangiocarcinoma; ECC: extrahepatic cholangiocarcinoma; NLR: neutrophil-lymphocyte ratio

|  | | **Univariate Logistic Regression** | |
| --- | --- | --- | --- |
|  |  | **OR (95% CI)** | **p-value** |
| **Age (years)** | Cont variable | 0.99 (0.94-1.05) | 0.919 |
| **Gender** | Male (vs Female) | 4.65 (1.31-16.45) | 0.017 |
| **ECOG Performance status** | ≥2 (vs 0-1) | 2.42 (0.81-7.27) | 0.115 |
| **Primary tumour site** | Ampullary cancer | 1 (Ref) | - |
|  | Cholangiocarcinoma | 5.60 (0.66-47.42) | 0.114 |
|  | Gallbladder | 1.33 (0.07-24.32) | 0.846 |
| **Primary tumour site** | Ampullary cancer | 1 (Ref) | - |
|  | ICC | 7.50 (0.84-66.86) | 0.071 |
|  | ECC | 3.43 (0.34-34.99) | 0.299 |
|  | Gallbladder | 1.33 (0.07-24.32) | 0.846 |
| **Primary tumour site (if cholangiocarcinoma)** | ECC (vs ICC) | 0.46 (0.12-1.79) | 0.260 |
| **Tumour differentiation** | Poorly-dif (vs Well/Mod) | 1.72 (0.50-5.89) | 0.386 |
| **Stage** | III-IV (vs I-II) | 1.50 (0.42-5.36) | 0.532 |
| **Ca19.9** | Cont variable | 1.001 (0.99-1.01) | 0.223 |
| **Albumin** | Cont variable | 0.95 (0.79-1.15) | 0.610 |
| **NLR** | Cont variable | 1.07 (0.95-1.21) | 0.246 |

OR: Odds ratio, 95% CI: 95% confidence interval; ECOG: Eastern Cooperative Oncology Group; ICC: intrahepatic cholangiocarcinoma; ECC: extrahepatic cholangiocarcinoma; NLR: neutrophil-lymphocyte ratio
